# Two Prevalent *Listeria ivanovii* subsp*. ivanovii* Clonal Strains With Different Virulence Exist in Wild Rodents and Pikas of China

**DOI:** 10.3389/fvets.2020.00088

**Published:** 2020-02-26

**Authors:** Lin Gan, Pan Mao, Huaying Jiang, Lu Zhang, Dongxin Liu, Xiaolong Cao, Yan Wang, Yiqian Wang, Hui Sun, Ying Huang, Changyun Ye

**Affiliations:** ^1^State Key Laboratory for Infectious Disease Prevention and Control, Collaborative Innovation Center for Diagnosis and Treatment of Infectious Diseases, National Institute for Communicable Disease Control and Prevention, Chinese Center for Disease Control and Prevention, Beijing, China; ^2^Hunan Provincial Key Laboratory for Special Pathogens, Institute of Pathogenic Biology, Medical College, University of South China, Hengyang, China; ^3^Department of Microbiology, School of Basic Medical Science, Guizhou Medical University, Guiyang, China; ^4^Institute of Infectious Disease, Guangzhou Eighth People's Hospital of Guangzhou Medical University, Guangzhou, China; ^5^Beijing Changping Institute for Tuberculosis Prevention and Treatment, Beijing, China

**Keywords:** *Listeria ivanovii* subsp. *ivanovii*, motility, metabolism, virulence, sequence type

## Abstract

*Listeria ivanovii* subsp. *ivanovii* is an intracellular bacterium distributed widely in nature, causing the listeriosis in ruminants and humans. Previous researches had isolated 116 strains of *L. ivanovii* subsp. *ivanovii* from wild rodents and pikas of different regions in China, and the predominant sequence types were ST1 and ST2. In this study, we first investigated the biological characteristics and virulence of these two clonal strains including motility, metabolism and virulence in cells and mouse model. The results demonstrated the ST1 strains exhibited motility, wide metabolic activity and hypervirulence, whereas the ST2 strains showed non-motility, relative lower metabolic activity and virulence. Considering the transmissible ability from wild rodents and pikas to ecological environment, the *L. ivanovii* subsp. *ivanovii* with potential pathogenicity to humans and ruminants should be monitored.

## Introduction

*Listeria ivanovii* and *Listeria monocytogenes*, which are the only two pathogenic in the genus *Listeria*, appear to be widely distributed in a variety of environments such as food, water, soil and feces of humans and animals (1–3). *L. monocytogenes* can cause various clinical manifestations such as septicemia, meningoencephalitis and diarrhea in human and animals. *L. ivanovii* can cause ruminant infection with similar pathological manifestations except meningoencephalitis, which is one of the characters of *L. monocytogenes* infection in ruminants and humans (2, 4, 5). In recent years, sporadic listeriosis caused by *L. ivanovii* in immunocompromised people have been reported (5, 6).

The two subspecies of *L. ivanovii*, which are subsp. *ivanovii* and subsp*. londoniensis*, had been reported (7, 8). They shared the same biochemical characteristics except the ability to utilize *N*-acetyl-β-D-mannosamine and ribose, which can be used to identify the subspecies (8). The strains of *L. ivanovii* subsp. *ivanovii* are sensitive to bacteriophages infection. While the strains of subsp. *londoniensis* seem to be resistant to bacteriophage infection except for a large virulent A511-like virus, and the LivCRISPR-1 which is a type II-A system may play a native role in phage defense (9, 10). *L. ivanovii* subsp. *ivanovii* caused the most of listeriosis in human and animals, and a previous study had found *L. ivanovii* subsp. *ivanovii* clinical strain G770 contains a type I restriction-modification system which may contribute to its hypervirulence (1, 6).

The pathogenicity of *L. ivanovii* mainly depends on pathogenicity island 1 (LIPI-1) and LIPI-2. LIPI-1 which also exists in *L. monocytogenes* harbors genes for a central virulence regulator (*prfA*), an actin polymerize surface protein (*actA*), a pore forming toxin (*hly*), a metalloprotease (*mpl*), and two phospholipases (*plcA* and *plcB*); LIPI-2 is specific for *L. ivanovii* and includes genes coding for a sphingomyelinase C (*smcL*) and several internalins (11). Except for *smcL* and *inlB1*, all LIPI-1 and LIPI-2 genes are regulated by *prfA* (11).

A total of 116 *L. ivanovii* subsp. *ivanovii* strains were isolated from intestinal contents of wild rodents from 12 provinces in China during 2014–2016 (12, 13). The strains belong to five sequence types (STs), and the predominant subtypes were ST1 (10/116, 8.62%) and ST2 (100/116, 86.21%). ST1 strains were isolated from feces of *Mus pahari, Apodemus chevrieri, Apodemus draco* and *Niviventer confucianus* in Tibet and Yunnan province, and their wide-range distribution suggests possible presence of bacteria transmission. ST2 strains were collected from intestinal contents of *Ochotona curzoniae* (pikas) and *Marmota himalayana* in Qinghai province. As the carriers of pathogens, wild rodents such as *Rattus rattus* and *Rattus norvegicus* have high reproducibility and free-living behavior, which enable them to play roles in ecological environment security by fecal-oral route (14, 15). Wild rodents and pikas that can carry pathogens such as H5N1 highly pathogenic avian influenza virus and shiga toxin-producing *Escherichia coli*, may play role in pathogenic *Listeri*a transmission among various species and even influence on ecological environment (16, 17). However, no data was reported about the biological characteristics and virulence of different ST strains of *L. ivanovii*. In this study, we first investigated the motility, metabolism and virulence of ST1 and ST2 strains from wild rodents of China.

## Materials and Methods

### Bacterial Strains, Plasmids, Cells, Media, and Culture Conditions

The *Listeria ivanovii* subsp. *ivanovii* reference strain PAM55 (ST1), ST1 strains (LIV037, LIV038, LIV039, LIV041, LIV042), and ST2 strains (LIV047, LIV048, LIV049, LIV050, LIV051) were cultured in brain-heart infusion (BHI) medium at 37°C. The ST1 strain LIV037 (ICDC-LIV037, CHPC 1. 5099) and ST2 strain LIV047 (ICDC-LIV047, CHPC 1. 5100) have been preserved in Center for Human Pathogen Collection (CHPC). Chemically competent cell Trans10 (Transgene Biotech) was used as the host strain for constitutive overexpression vector PIMK2 and cultured in Luria-Bertani (LB) medium at 37°C. Bacterial motility was tested on semisolid medium (BHI broth containing 0.3% agar), and BHI plate containing 5% lecithin was used to test the lecithin utilization. The human colon carcinoma cells (Caco-2) and bovine kidney cells (MDBK) were cultured at 37°C under 5% CO_2_ in DMEM and MEM medium containing 10% FBS, respectively.

### Average Nucleotide Identity (ANI) Analysis

The 28 strains (Table S1) of *Listeria* with genome sequences available online were chosen for ANI analysis (18). The unweighted pair group method with arithmetic mean method (UPGMA) based dendrogram was constructed by Software Bionumerics.

### Motility Assay

The *L. ivanovii* subsp. *ivanovii* strains were inoculated in BHI semisolid medium at 25°C and scanned by Transmission electron microscope.

### Relative Expression Quantity of Genes

Real time-PCR was used to determine the relative expression quantity of LIPI-1 genes and motility related genes (*gmaR, flaA*). RNA was extracted from log-phase bacteria by Trizol (Ambion), then reverse transcribed by PrimeScript^TM^ reagent kit with gDNA Eraser (TaKaRa) according to manufacturer's instructions. The acquired cDNA was measured by Real-time PCR using SYBR® Premix Ex Taq^TM^ II (TaKaRa) and calculated by 2_T_^−ΔΔC^ method (19). Each RT-PCR was conducted in the 20 μL aliquot of running buffer containing 50–100 ng cDNA, annealing temperature was 60°C. The relative expression quantity was normalized by reference gene *16SrDNA*. Primers were shown in Table S2.

### Recombination Plasmids Construction and Electro-Transformation

To construct recombination plasmids, the DNA fragments were obtained by PCR using primers in Table S2 and purified DNA Gel Extraction Kit (Takara). The purified products were cloned into restriction enzyme cutting sites of vector PIMK2 by T4 ligase (NEB) cloning. Recombination plasmids were introduced into competent cells of LIV047, and the electroporation conditions were 2.5 KV, 25 μF, 400 Ω. Then the recombination strains were screened from BHI plates containing 50 μg/mL kanamycin.

### *In vitro* Growth Assay and Phenotype MicroArray (PM) Analysis

For *in vitro* growth assay, the log-phase bacteria were transferred to BHI broth with 1:100 dilution and cultured for 72 h, and the OD600 values were determined by Bioscreen C microbiology reader per hour.

The Carbon source (C-source, PM01-02) and Nitrogen source (N-source, PM03, 06-08) utilization tests were performed as described in Biolog instructions of Phenotype MicroArray Procedures for *B. subtilis* and other GP Bacteria (20). The areas of growth curves (72 h) of *L. ivanovii* subsp. *ivanovii* strains were compared.

### Identification of Virulence Related Metabolism Genes of Differential Metabolism Phenotypes Between ST1 and ST2 Strains

The metabolism genes related with virulence were systemic summarized by previous studies (21–24). The similarities of amino acid between the tested strain and PAM55 were calculated by software MegAlign.

### Invasion and Intracellular Growth Assays in Caco-2 and MDBK Cells

Human-derived Caco-2 (2 × 10^5^) and bovine-derived MDBK cells (1 × 10^5^) were incubated in 24-well plates the day before invasion with the log-phase bacteria at multiplicity of infection (MOI) 100. After an hour infection, cells were incubated for 1 h in DMEM (for Caco-2) or MEM (for MDBK), both containing 50 μg/mL gentamicin. Then the bacteria were released from cells through adding PBS containing 0.1% Triton-X100 at 1, 3, 6, and 12 h post-infection, and incubated on BHI plate for colony counting.

### Cytotoxicity Assays in Caco-2 and MDBK Cells

Cytotoxicity assay (Promega) was used to measure cells lactate dehydrogenase (LDH) release that indicates the cells membrane damage. Caco-2 (4 × 10^4^) and MDBK cells (2 × 10^4^) were incubated in 96-well plates the day before infection with the bacteria at MOI 100. After 3 h of incubation, the LDH in cell supernatant was measured according to manufacturer's instructions (25).

### Mouse Infection and Examination of Organs

Experimental procedures involving mouse model were approved by Welfare and Ethical Review in Animal Experimentation of National Institute for Communicable Disease Control and Prevention, China CDC, and carried out by the licensed individual with a license number of 2017-0021. Six to seven weeks old female BALB/c mice (Charles River) were used in the assays and conducted as previous described with some modifications (26–28). For 50% lethal doses (LD_50_) determinations, groups of 10 mice were injected intraperitoneally with 5-fold dilutions of log-phase bacteria (5 × 10^8^, 1 × 10^8^, 2 × 10^7^, 4 × 10^6^, and 8 × 10^5^ CFU in 200 μL PBS) and monitored for 7 days, mice of control group were injected with 200 μL PBS.

The infection doses were 10-fold lower than LD_50_ (1.70 × 10^6^ CFU) of the reference strain PAM55 in subsequent experiment analyses. Five mice were euthanized for weighing and analysis of bacterial loads in organs (liver, spleen, lung, kidney, small intestine, and brain) each for seven consecutive days, and histopathological changes of organs were analyzed at 1, 4, and 7 days post-infection (dpi). Each organ was divided into two parts, one for hematoxylin-eosin (HE) staining, the rest was homogenized in PBS containing 0.5% Triton-X100 and 10-fold diluted for colony counting with three replicates on BHI plate. In addition, small intestine tissues were rinsed by DMEM and incubated in DMEM containing 100 mg/L gentamicin for 1 h to kill extracellular bacteria before HE staining and colony counting.

### Identification of Virulence Genes

The genome sequence of ST1 strain and ST2 strain were analyzed on website of Virulence factors of Pathogenic Bacteria (http://www.mgc.ac.cn/VFs/main.htm), and the similarities of amino acid were calculated by software MegAlign (29). PAM55 was used as reference strain.

### Statistical Analyses

Data of curves were presented as mean ± SD and subjected to repetitive measurement deviation analysis. Other results were presented as mean ± SD and subjected to Two-way ANOVA and Student's test. Differences were judged statistically (SPSS 20.0) significant at *P* < 0.01 or 0.05. Software Graphpad 7.0 was used to plot figures.

## Results

**Figure 1:**
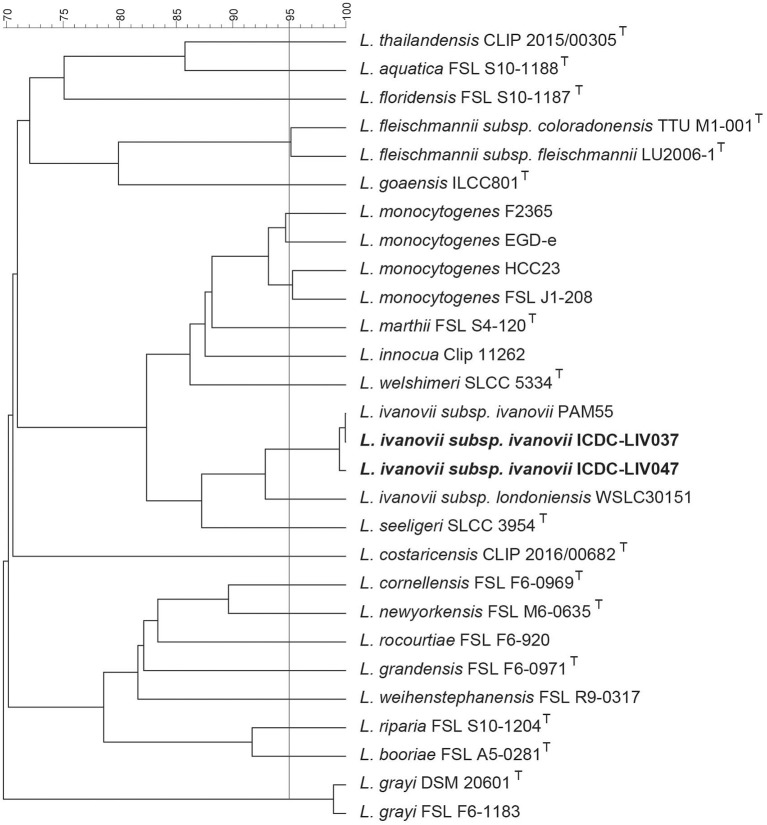
The dendrogram of UPGMA clustering based on ANI. The 20 species of *Listeria* including *L. ivanovii* were used to construct the dendrogram. The vertical bar represents the 95% ANIb that correlates with the 70% DNA-DNA hybridization threshold (30). The reference strain PAM55, ST1 strain LIV037, and ST2 strain LIV047 were formed an independent clade respectively, and the ANI between them were larger than 99%.

The 20 species of *Listeria* including *L. ivanovii* were used to construct a UPGMA based dendrogram (Figure 1). The reference strain PAM55, ST1 strain LIV037, and ST2 strain LIV047 formed an independent clade and the ANI between them was larger than 99% (30).

*In vitro* growth curve, motility assay and cellular infection were performed to investigate biological characteristics of all ST1 and ST2 strains. Similar characteristics of ST1 strains (LIV037, LIV038, LIV039, LIV041, LIV042) and ST2 strains (LIV047 LIV048, LIV049, LIV050, LIV051) were obtained, thus LIV037 (ST1) and LIV047 (ST2) with genomic information were chosen for further analysis, including transmission electron microscope scanning, Phenotype MicroArray analysis, mouse assay, and relative expression quantity of LIPI-1 genes and motility related genes.

### Motility Analysis

**Figure 2:**
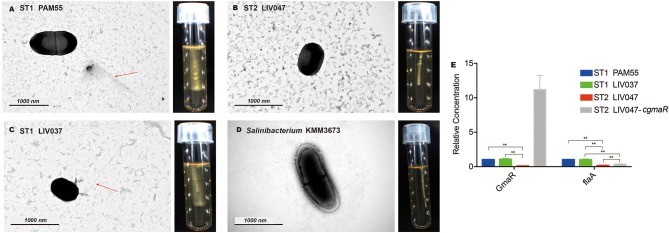
**(A–D)** Transmission electron micrographs and motility assays of *L. ivanovii* subsp. *ivanovii* at 25°C. The red arrow denotes the flagella. **(E)** Relative quantification of flagellar-related gene transcriptional levels. The transcriptional level of each gene was normalized to that of *16S rDNA*. Bars represent the mean of three replicates mean ± SD. **p < 0.01.

The reference strain PAM55 and ST1 strains demonstrated cloudy growth and swarming motility around the stab in semisolid medium at 25°C. No evidence of motility was observed in all ST2 strains (Figures 2A–D). The flagellum was observed in ST1 strain LIV037 using transmission electron microscope, but not in ST2 strain LIV047. Compared with strain PAM55, 24 motility related genes in LIV047 strain have non-synonymous mutations and/or indels (Table S3). Notably, in ST2 strains, a premature stop codon (PMSC) was identified in *gmaR*, which was important in regulating expression of flagellar genes such as *flaA*, and the relative expression levels of *gmaR* and *flaA* in LIV047 were significantly lower than that in PAM55 and LIV037 (*P* < 0.01) using real-time PCR (Figure 2E). However, a recombinant strain LIV047-*cgmaR* with full length *gmaR* in constitutive overexpression vector still showed non-motility (data not shown), and the expression levels of *flaA* was still significantly (*P* < 0.01) lower than ST1 strains.

### Metabolism Phenotypes

**Figure 3:**
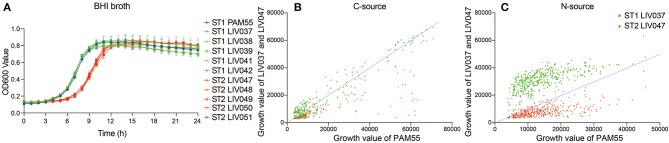
**(A)** Growth curve of *L. ivanovii* subsp. *ivanovii* strains in BHI broth. The growth of each strain was exhibited by different color as legend, bars represent the mean of five replicates mean ± SD. **(B)** Phenotype MicroArray data of *L. ivanovii* subsp. *ivanovii* strains in 192 carbon-sources. **(C)** Phenotype MicroArray data of *L. ivanovii* subsp. *ivanovii* strains in 384 nitrogen-sources. The growth values were the area under growth curve, and the dotted line represents the growth of reference strain PAM55.

The proliferation ability of strains was compared by growth curve in BHI broth. Significantly different growth rate (*P* < 0.01) between ST1 and ST2 strains were shown in Figure 3, and exponential phase of ST2 strains lagged behind ST1 strains by about 3 h, and ST1 strains shared similar growth characteristics with PAM55 (Figure 3A). The utilization of C-source and N-source were tested by Phenotype MicroArray system (Figures 3B,C). The comparison of growth curves in 192 C-sources (PM01-02) showed that ST2 strain LIV047 had a lower metabolic activity. No significant difference was found between ST1 strain PAM55 and LIV037 regarding C-sources utilization. In the 384 N-sources (PM03, 06-08) utilization assays, ST1 strain LIV037 grew better than other strains, and ST2 strain LIV047 also exhibited a lower metabolic activity.

In addition, 11 carbon and nitrogen differential utilization related proteins were analyzed, and the similarities of proteins in LIV037 and LIV047 refer to PAM55 were displayed in Table S4. Most proteins in LIV037 and LIV047 have bits of non-synonymous mutations (similarity >98%). However, glycerol utilization related protein *glpF* had 105 non-synonymous mutations, and ethanolamine utilization related gene *eutA, eutB*, and *eutC* were absent in the ST2 strain LIV047, which was consistent with the result of glycerol and ethanolamine utilization in Phenotype MicroArray test.

### Virulence Analysis

**Figure 4:**
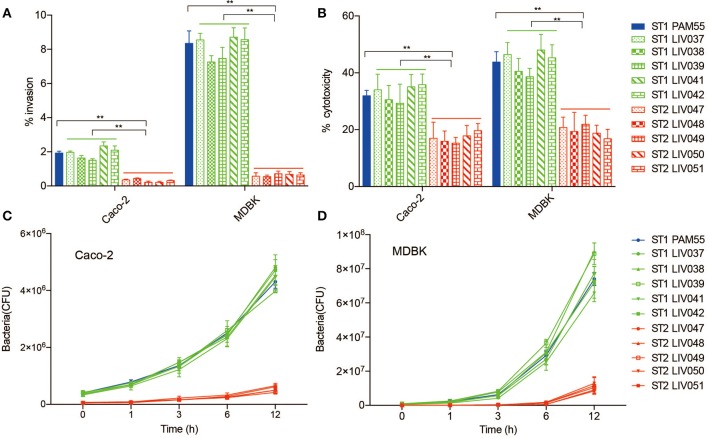
Virulence analysis of *L. ivanovii* subsp. *ivanovii* strains in MDBK and Caco-2 cells. Invasion rate **(A)** and cytotoxicity **(B)** were measured at 1 and 3 h post-infection. Intracellular growth **(C,D)** was recorded for 12 h post-infection. Bars represent the mean of nine replicates mean ± SD. **p < 0.01.

The ability of internalization, bacterial cytotoxicity, and intracellular growth were evaluated in both Caco-2 and MDBK host cells. Significantly (*P* < 0.01) higher invasion ability and cytotoxicity were found for ST1 strains than ST2 strains in both Caco-2 and MDBK cells (Figures 4A,B). For intracellular growth assay, ST1 strains exhibited significantly (*P* < 0.01) more efficient growth rate than ST2 strains, and the rapid growth appeared obviously from 3 to 6 h post-infection in Caco-2 and MDBK cells (Figures 4C,D).

**Figure 5:**
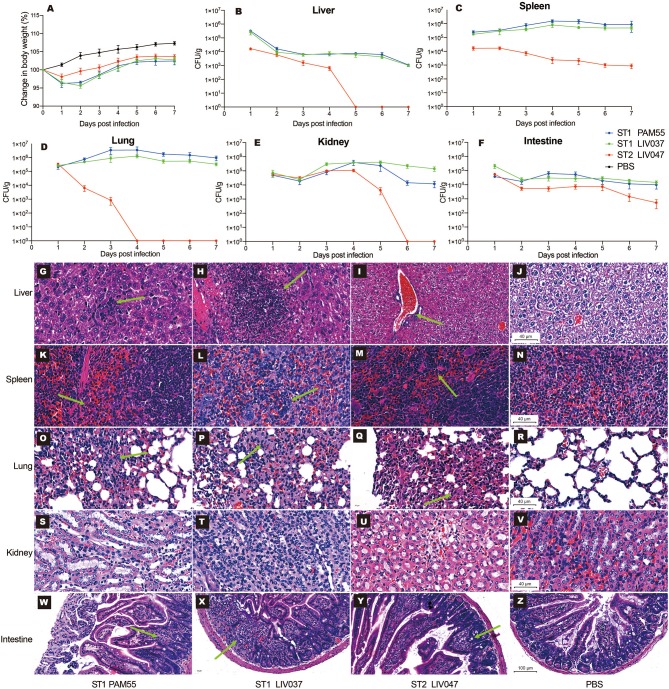
Virulence analysis of *L. ivanovii* subsp. *ivanovii* strains in mouse model. Change in body weight **(A)** and bacterial loads in the liver **(B)**, spleen **(C)**, lung **(D)**, kidney **(E)**, and intestine **(F)** were recorded for 7 days. Bars represent the mean of five replicates mean ± SD. Histopathologic analysis of organs in 4 dpi were shown in **(G–Z)**. **(G–V)** were captured under the 40x microscope, and **(W–Z)** were captured under the 20x microscope. The green arrows denote the pathologic injuries such as neutrophil infiltration, hemorrhage, alveolar septum widened and granuloma.

The virulence of ST1 and ST2 strains was examined *in vivo* using BALB/c mouse model of intraperitoneal infection. The LD_50_ of PAM55, LIV037, and LIV047 to BALB/c mice were 1.70 × 10^7^ CFU, 2.00 × 10^7^ CFU, and 2.24 × 10^8^ CFU, respectively. Then the virulence of strains was further evaluated in mice infected by ST1 and ST2 strains (1.70 × 10^6^ CFU), changes in body weight and bacterial loads in organs were measured for 7 days post-infection. No significant difference (*P* > 0.05) of maximum weight loss was observed between mice infected with PAM55 (1 dpi, 3.74% of original weight) and LIV037 (2 dpi, 4.38% of original weight); however, significantly less weight loss (1 dpi, 1.90% of original weight) was observed in mice infected with LIV047 than that of PAM55 (*P* < 0.05) and LIV037 (*P* < 0.01) (Figure 5A). Bacterial loads in liver, spleen, lung, kidney, intestine, and brain of mice were measured daily. No bacterium was detected by colony counting and real-time PCR (genus-specific gene *prs*) in the brain tissues of all mice (data not shown), the bacterial loads in other organs of mice of LIV047 infected group were significantly (*P* < 0.01) less than that of PAM55 and LIV037 infected groups. The bacteria were persistent in the liver, spleen, lung, kidney, and intestine of the *L. ivanovii* infected mice through 7 days except for the LIV047 group in which bacteria disappear at 5 dpi in liver, 4 dpi in lung and 6 dpi in kidney (Figures 5B–F).

To evaluate and compare pathological injury in mice organs, mice were sacrificed and examined at 1, 4, and 7 dpi. Consistent with the bacterial loads, PAM55 and LIV037 strains showed more severe pathological injuries to mice than LIV047. In the mice with PAM55 and LIV037 infection, liver cell degeneration and necrosis with lots of neutrophils aggregation emerged in livers at 1 dpi, and severe necrosis appeared at 4 dpi, which lasted until 7 dpi for PAM55 group, while only a few of neutrophils were observed for LIV037 group. In contrast, the necrosis only appeared in liver tissue at 1 dpi for LIV047 group, and few inflammatory cells existed at 4 and 7 dpi (Figures 5G–J, Figure S1). Marked neutrophil infiltration were observed in red pulp of the spleen at 1, 4, and 7 dpi for mice of PAM55 and LIV037 infected groups, but 1 dpi for LIV047 group with fewer neutrophils at 4 and 7 dpi (Figures 5K–N, Figure S2). Wild inflammation including hemorrhage, alveolar septum widened, collapsed alveoli and inflammatory cells infiltrate were observed in the lungs of mice infected by PAM55, LIV037, and LIV047 at 1 and 4 dpi; same character also lasted at 7 dpi for LIV037 and PAM55, but not for LIV047 (Figures 5O–R, Figure S3). No histopathology change was observed in kidney of all three groups (Figures 5S–V, Figure S4). For small intestine, the granuloma was observed in the mice infected by PAM55 and LIV037 strain at 4 and 7 dpi, and by LIV047 strain at 7 dpi (Figures 5W–Z, Figure S5).

**Figure 6:**
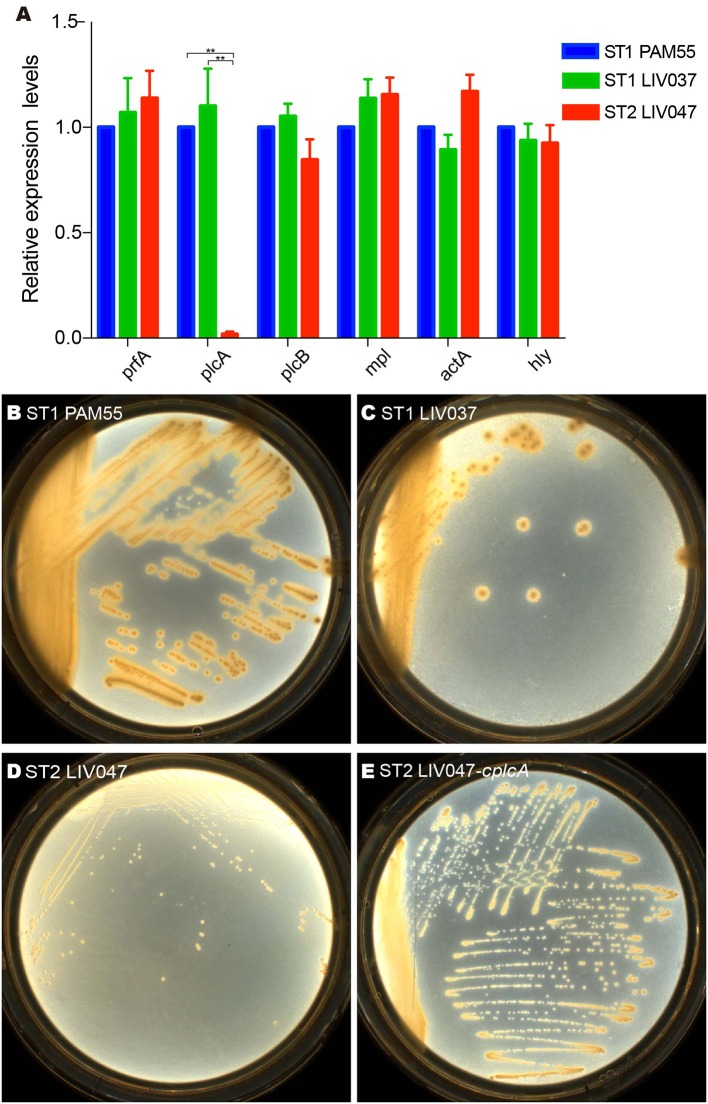
**(A)** Relative quantification of LIPI-1 gene cluster transcriptional levels. The transcriptional level of each gene was normalized to that of *16SrDNA*. Bars represent the mean of three replicates mean ± SD. **P < 0.01. **(B–E)** Colony morphology of *L. ivanovii* subsp. *ivanovii* strains on BHI plate containing 5% lecithin.

The virulence genes of strains were analyzed. ST1 strain LIV037 and ST2 strain LIV047 were positive for LIPI-1, LIPI-2 and other 26 virulence genes, and LIV037 has more similarity to PAM55 than LIV047 (Table S5). Compared to PAM55, most virulence factors in ST1 and ST2 strains have bits of non-synonymous mutations (similarity >95%). However, in LIV047, deletions and several non-synonymous mutations were identified in *actA* (119 amino acids deletion which located on an unknown domain and 15 non-synonymous mutations), *inlB2* (309 amino acids deletion which located on the bacterial adhesion/invasion protein N-terminal and Gly-Tryp dipeptide domain, and 3 non-synonymous mutations) and *agrC* (237 amino acids deletion which located on the hyaluronan mediated motility receptor N-terminal, sensor kinase SpoOB-type and alpha-helical domain). In addition, LIV037 and LIV047 shared similar expression levels of six virulence genes in LIPI-1 with PAM55 strain (Figure 6A), but expression levels of *plcA* gene coding 1-phosphatidylinositol phosphodiesterase in LIV047 was significantly lower than that of PAM55 and LIV037 strains (*P* < 0.01). Four amino acid changes (S83A, K235E, A245T, and T310R) in *plcA* were identified for LIV047 strain compared with PAM55 strain. Then a recombinant strain LIV047-*cplcA* containing the same *plcA* in PAM55 strain was constructed, but no significant difference of cytotoxicity was found between LIV047-*cplcA* and LIV047 strains (*P* > 0.05, data not shown). However, white halos around the colonies on the BHI plate containing 5% lecithin was observed for the recombinant strain LIV047-*cplcA* but not for ST2 strains including LIV047 (Figures 6B–E).

## Discussion

*Listeria ivanovii* was distributed widely in nature and has potential to cause listeriosis in ruminants and even immunocompromised humans (5, 6). Previous studies have noted that wild rodents and pikas in China carried various STs of *L. ivanovii* subsp. *ivanovii*, and the predominant sequence types were ST1 and ST2 (12, 13). ST1 strains were collected from five species of wild rodents in Tibet and Yunnan province, which reveals the potential ability of ST1 clonal strains to spread cross species and regions (13). However, the ST2 strains were only found in Yushu Tibetan autonomous prefecture of Qinghai province. This is the first study to characterize two different clonal STs of *L. ivanovii* subsp. *ivanovii* on the aspects of motility, metabolism and virulence.

Flagellar motility enhances extracellular survival and colonization in host during *Listeria* infection (31–34). *L. ivanovii* subsp. *ivanovii* ST2 strains were the first identified non-motility subtype in this study. Previous report noted gene *gmaR* is the key thermometer which controls the temperature-dependent transcription of motility genes such as *flaA* in *L. monocytogenes*, and a premature stop codons (PMSC) contributed to the lack of Tetratricopeptide Repeat and Anti-Repressor domains was found in *gmaR* of ST2 strains (35). However, constitutive expression of complete *gmaR* did not recover the motility and expression level of *flaA* in ST2 strains. The changes of other motility related genes or other unknown mechanism involved in motility may probably attribute to the non-motility of ST2 strains.

The virulence analysis strongly supports the more virulent property of ST1 strains compared to ST2 strains. Transcription levels of six genes (*actA, hly, mpl, plcA, plcB, and prfA*) in LIPI-1 were measured for the two different clonal strains, of which *plcA* in ST2 strain was significantly lower than that of ST1 strain. Gene *plcA* was *prfA*-regulated, and cooperate with *plcB* to lyse the phagocytic vacuole membrane (36). By utilizing phosphatidylcholine, *plcA* contributes to the formation of white halo around colony in lecithin plate, and has been used as a virulence marker to distinguish the pathogenic and nonpathogenic *Listeria* to humans (37, 38). The low transcription level of *plcA* in ST2 strain corresponds to lose of white halo. In addition, previous carbon-source regulation research found that cellobiose could repress the transcription of *plcA* independent of *prfA* pathway (39). Cellobiose could not be utilized by ST2 strain in metabolism assay, its accumulation in organism probably affect the expression level of *plcA*. We found complete *plcA* enabled LIV047 strain to acquire phenotype of white halo on plate, but no significant difference of cytotoxicity was observed between LIV047-*cplcA* and LIV047 in cultured cells. Other virulence genes analysis found deletion mutations and lots of non-synonymous mutations which could potentially affect genes functions existed in *actA, inlB2*, and *agrC* of ST2 strain LIV047, these virulence genes are important for pathogenicity of *Listeria* (40–42). Whether it is the reason for the lower virulence of ST2 strains needs to be proved.

*Listeria* has its specific metabolic adaptations to promote colonization in eukaryotic cells, and metabolic pathways of glycerol, glutamine, amino acids, and peptides are crucial for the evasion of innate immunity, intracellular replication and survival (22, 24). We found ST1 strains exhibited higher carbon and nitrogen metabolic activity and growth rate in nutrient rich medium than ST2 strains, which was possibly associated with the wide distribution of ST1 strains in China.

The capacity of pathogens to efficiently utilize various nutrient sources in nature is crucial for its increased survival and expanded niche range in hosts and ecological environments (20). Glycerol utilization related genes such as *glpF* and *glpK* were up-regulated in host cells and the intestine of mice (43, 44). However, the *glpF* of ST2 strain has only 54.94% similarity to ST1 strain PAM55. Ethanolamine and branched chain amino acids (BCAAs) were required for intracellular replication, promote *Listeria* lipid membrane homeostasis and resistance to intracellular stresses (45, 46). We found that *eutABC* genes required to ethanolamine utilization were absent in ST2 strain. Lower metabolic activity of utilizing glycerol, ethanolamine, amino acids, and peptides in ST2 strain are consistent with the lower virulence in cells and mouse models in this study. Whether the mutations or transcriptional levels of these carbon and nitrogen utilization related genes affect the virulence ST2 strain are unknown.

All ST2 strains were isolated from Qinghai-Tibetan, which is the highest altitude in the world and comprises unique ecological environment with intense ultraviolet radiation and poor carbon and nitrogen sources. Whether the extreme ecological environment contributed to the variation in genes especially for those metabolism related genes needs to be further explored. Besides, ST2 strain's carriage is high in wild rodents and pikas, possibly because the relative lower virulence of ST2 strains enables them to colonize the gut for a long period of time, further studies are needed to verify this hypothesis.

In conclusion, we first reported the phenotypic characteristics and virulence of two prevalent *L. ivanovii* subsp. *ivanovii* clonal strains in wild rodents and pikas of China. They varied from motility, metabolism to virulence in cells and mouse model. The ST1 strains exhibited motility, wide metabolic activity and hypervirulence, whereas the ST2 strains showed non-motility, relative lower metabolic activity and virulence. Previous study has noted that bovine trophoblastic cells are susceptible to both *L. monocytogenes* and *L. ivanovii* infection, which may explain the abortions and reproductive failures caused by *L. ivanovii* infection in cattle (47). Considering the transmissible ability from rodents to ecological environment, the *L. ivanovii* subsp. *ivanovii* strains especially for ST1 strains with potential pathogenicity to humans and ruminants should be monitored. Further studies should focus on the specific mechanism of adaption to the different habitats and virulence of the two clonal strains of *L. ivanovii* subsp. *ivanovii*.

## Data Availability Statement

All datasets generated for this study are included in the article/Supplementary Material.

## Ethics Statement

The animal study was reviewed and approved by National Institute for Communicable Disease Control and Prevention, Chinese Center for Disease Control and Prevention.

## Author Contributions

LG and CY designed the research study and wrote the manuscript. LG, PM, HJ, LZ, DL, XC, YaW, YiW, HS, and YH performed experiments. LG analyzed the data.

### Conflict of Interest

The authors declare that the research was conducted in the absence of any commercial or financial relationships that could be construed as a potential conflict of interest.

## Supplementary Material

Figure S1 Histopathologic analysis of liver in mice infected with *L. ivanovii* subsp. *ivanovii* strains. Images were captured under the 40x microscope.

Figure S2 Histopathologic analysis of spleen in mice infected with *L. ivanovii* subsp. *ivanovii* strains. Images were captured under the 40x microscope.

Figure S3 Histopathologic analysis of lung in mice infected with *L. ivanovii* subsp. *ivanovii* strains. Images were captured under the 40x microscope.

Figure S4 Histopathologic analysis of kidney in mice infected with *L. ivanovii* subsp. *ivanovii* strains. Images were captured under the 40x microscope.

Figure S5 Histopathologic analysis of intestine in mice infected with *L. ivanovii* strains. Images were captured under the 20x microscope.

Table S1 Strains used in this study.

Table S2 Primers used in this study.

Table S3 Amino acid changes of motility related proteins in ST2 strain LIV047.

Table S4 Percentage identities of metabolism related proteins in ST1 strain LIV037 and ST2 strain LIV047 referring to PAM55.

Table S5 Percentage identities of virulence related proteins in ST1 strain LIV037 and ST2 strain LIV047 referring to PAM55.
