# Supplementary figures and images for: Two Prevalent Listeria ivanovii subsp. ivanovii Clonal Strains With Different Virulence Exist in Wild Rodents and Pikas of China
Source: Front Vet Sci. 2020 Feb 26;7:88. doi: 10.3389/fvets.2020.00088 (PMC7054220; doi:10.3389/fvets.2020.00088)

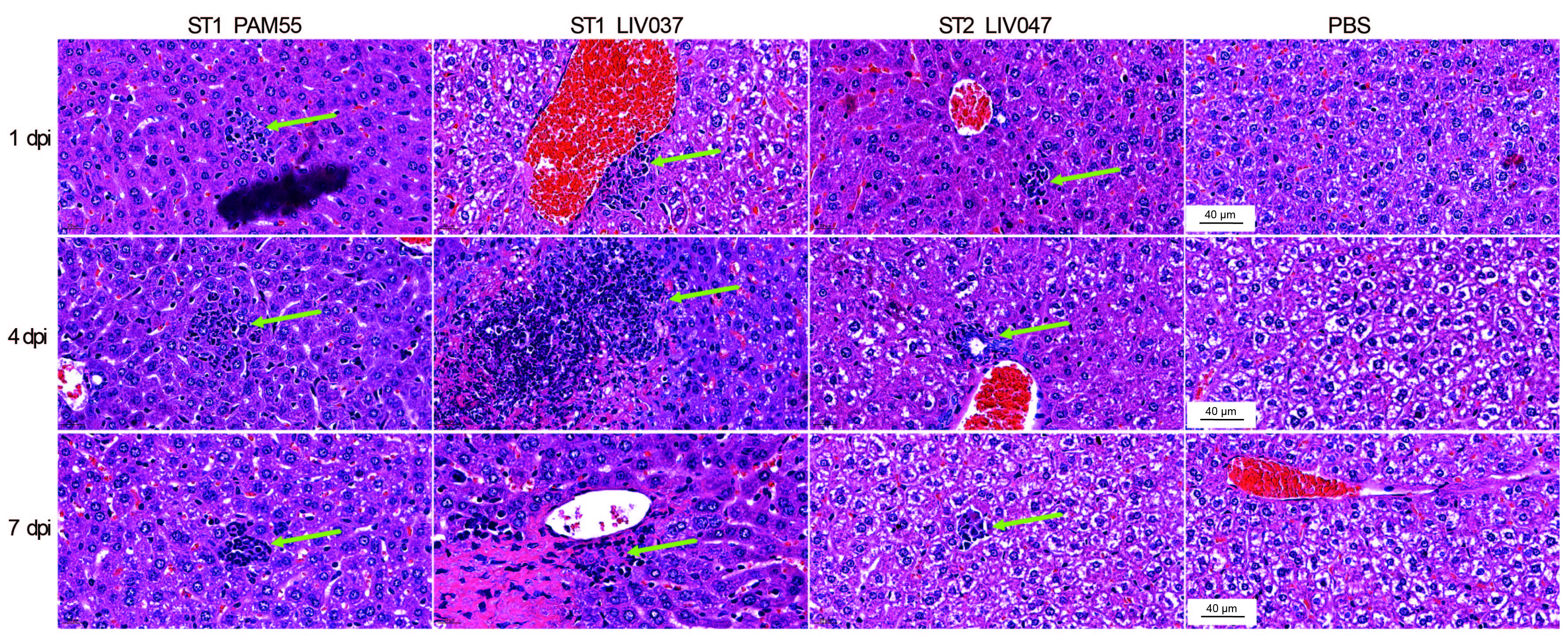

Supplement: Figure S1 — Histopathologic analysis of liver in mice infected with L. ivanovii subsp. ivanovii strains. Images were captured under the 40x microscope. [file Image_1.jpeg]

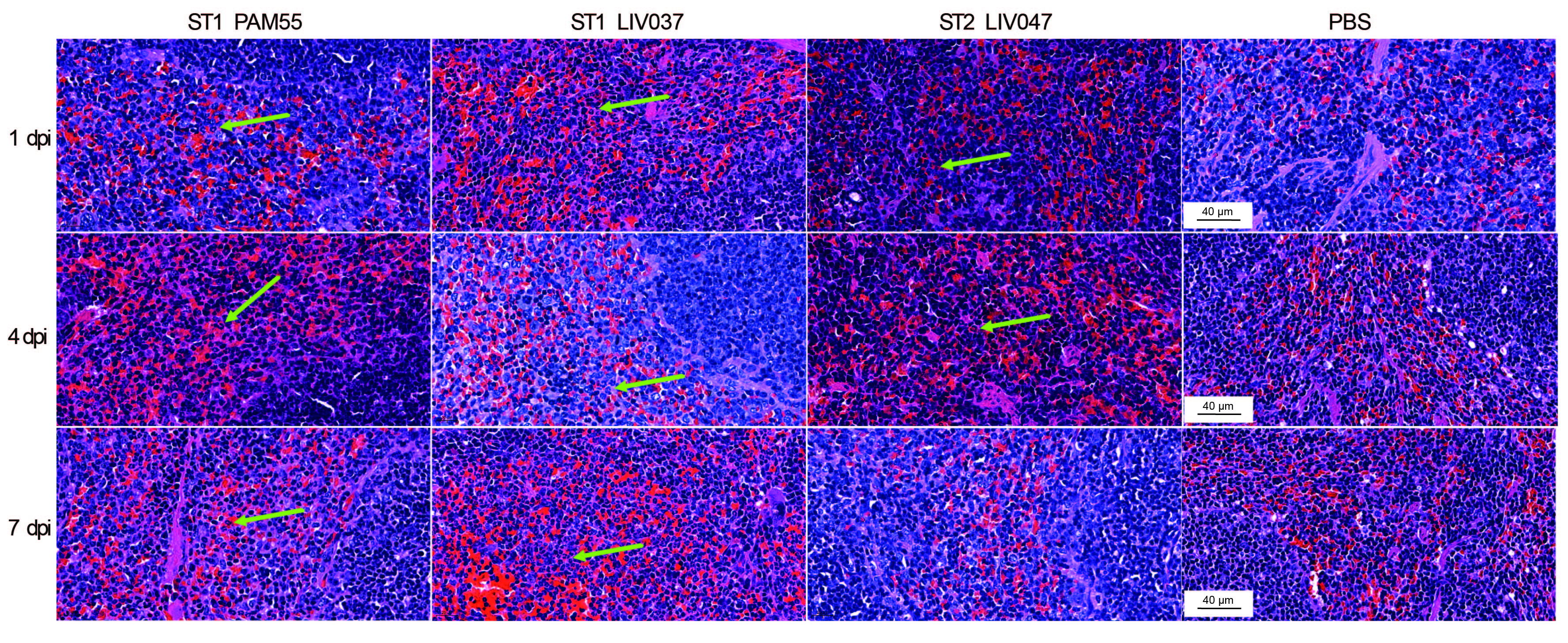

Supplement: Figure S2 — Histopathologic analysis of spleen in mice infected with L. ivanovii subsp. ivanovii strains. Images were captured under the 40x microscope. [file Image_2.jpeg]

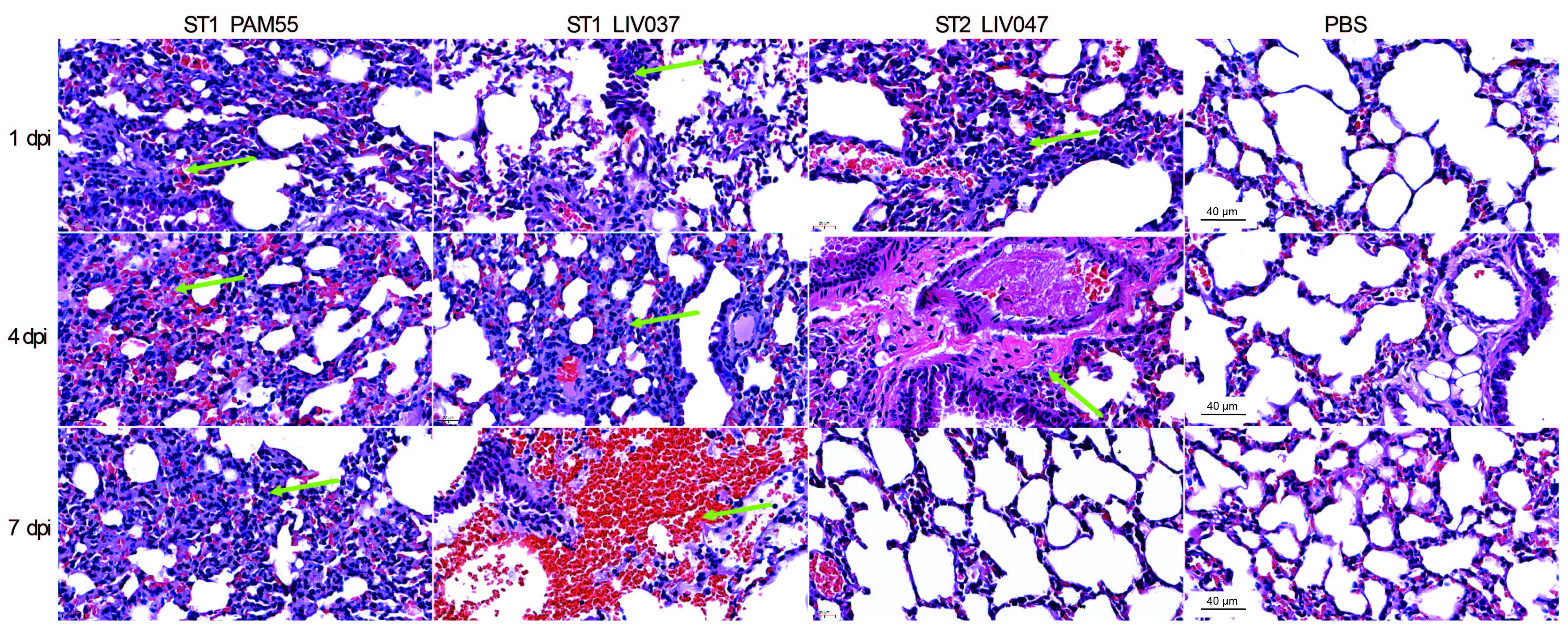

Supplement: Figure S3 — Histopathologic analysis of lung in mice infected with L. ivanovii subsp. ivanovii strains. Images were captured under the 40x microscope. [file Image_3.jpeg]

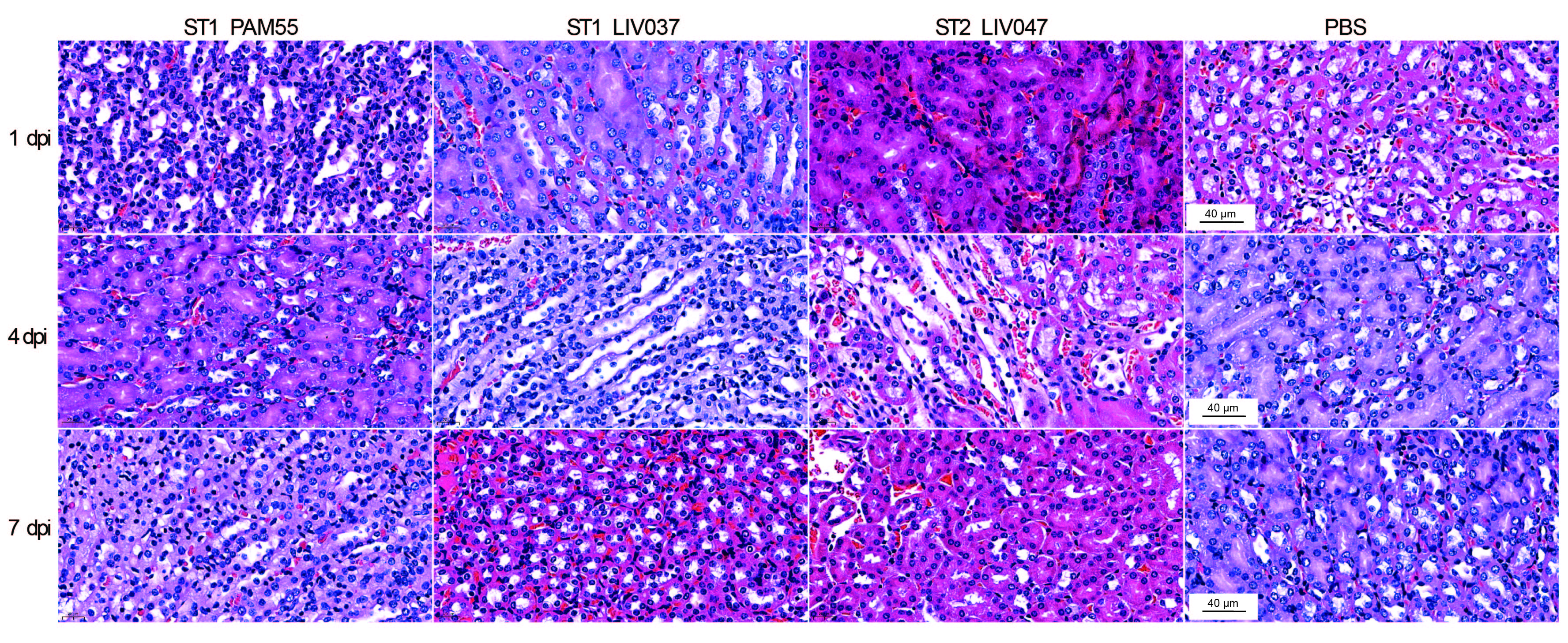

Supplement: Figure S4 — Histopathologic analysis of kidney in mice infected with L. ivanovii subsp. ivanovii strains. Images were captured under the 40x microscope. [file Image_4.jpeg]

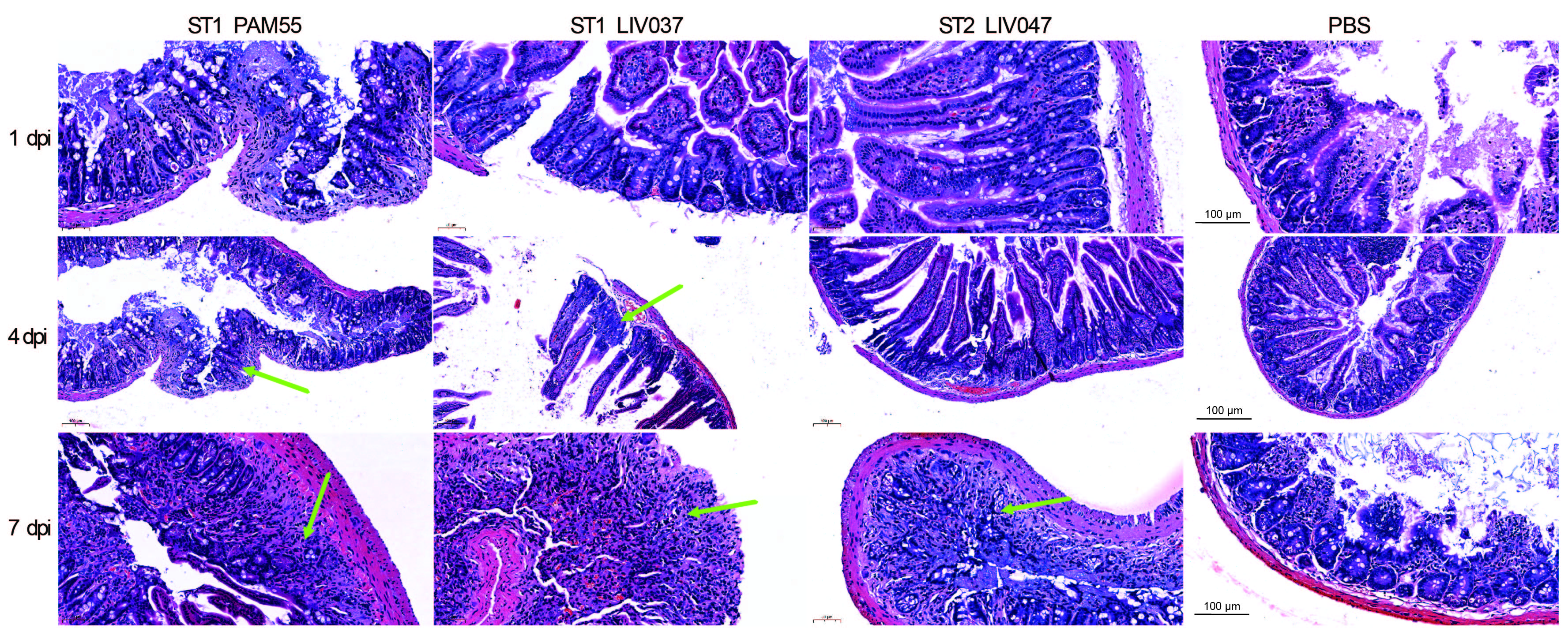

Supplement: Figure S5 — Histopathologic analysis of intestine in mice infected with L. ivanovii strains. Images were captured under the 20x microscope. [file Image_5.jpeg]
